# Improving motor skills and psychosocial behaviors in children with autism spectrum disorder through an adapted judo program

**DOI:** 10.3389/fpsyg.2022.1067310

**Published:** 2022-11-29

**Authors:** Jose Morales, Emanuela Pierantozzi, David H. Fukuda, Vanessa Garcia, Miriam Guerra-Balic, Marta Sevilla-Sánchez, Eduardo Carballeira

**Affiliations:** ^1^Faculty of Psychology, Education Sciences and Sport Blanquerna, Ramon Llull University, Barcelona, Spain; ^2^Department of Neuroscience, Rehabilitation, Ophthalmology, Genetics, Maternal and Child Health, University of Genoa, Genoa, Italy; ^3^School of Kinesiology and Physical Therapy, University of Central Florida, Orlando, FL, United States; ^4^Department of Physical Education and Sports, Faculty of Sport Sciences and Physical Education, University of A Coruña, Oleiros, Spain

**Keywords:** combat sports, ASD, TGMD, GARS, intellectual disabilities, adapted sports

## Abstract

**Introduction:**

This study aimed to investigate the long-term effects of an adapted judo program on the motor skills and psychosocial abilities of children with Autism Spectrum Disorder (ASD).

**Methods:**

All participants had been diagnosed with ASD and were assessed twice, one time at the start of the intervention and again 6 months later, with the Test of Gross Motor Development (TGMD-3) and the Gilliam Autism Rating Scale-Third Edition (GARS-3). A one-way repeated measures MANOVA was carried out in order to evaluate these assessments, and a mediation analysis was done to determine the relationship between them.

**Results:**

The experimental group significantly improved (*p* < 0.05) from the pre-test to the post-test for several subtests of the TGMD-3 and the GARS-3.

**Conclusion:**

The study shows that participation in an adapted judo program clearly helps to improve the motor skills and psychosocial behaviors of children with ASD.

## Introduction

Autism spectrum disorder (ASD) is a neurodevelopmental disorder with an unknown cause that manifests in difficulties and barriers associated with social communication and repetitive and stereotyped behaviors (American Psychiatric Association, 2013). There has been a recent increase in the prevalence of ASD among children, as in the mid-1990s it was thought to affect 11.6 out of 1,000 people in this age group (Baird et al., 2006), while more recent studies have put this figure as high as 18.5 per 1,000 (Maenner et al., 2020).

Several researchers have shown that people with ASD experience difficulties in social interactions and communication (Jones and Frederickson, 2010; Wilmshurst and Brue, 2018), leading to problems in their relationships with others. They are often characterized by a lack of motivation, resistance to change, and difficulties following social rules (Habib et al., 2018). Additionally, people with ASD often have problems with motor skills (Colombo-Dougovito and Block, 2019; Crucitti et al., 2020), affecting their ability to interact with others including successful social participation (Bodison, 2015).

It is yet unclear whether these motor difficulties are a direct consequence of the disorder itself or whether they emerge from an amalgam of other factors, such as a lack of opportunities to practice these abilities or a lack of motivation. It is clear, though, that children with ASD tend to lag behind their peers without the disorder when it comes to motor development (Pan et al., 2017; Sansi et al., 2021). This means that it is sometimes difficult for children with ASD to participate in activities that require motor skills that would otherwise be typical for their age groups, thereby further limiting their opportunities for social interaction.

Substantial evidence exists detailing these motor limitations. Studies have found that children with ASD perform poorly compared to their peers across various motor domains including balance, postural control and overall coordination (Downey and Rapport, 2012), gait disturbances, lateral movement and fine motor skills (Kaur et al., 2018). During early infancy, the differences in motor development between children with and without ASD are less pronounced. However, starting at about 18 months, the gap becomes more apparent, with children with ASD displaying less interest in play and spontaneous games (Serrada-Tejeda et al., 2021). This could further affect their motor and sensory development and, in turn, lead them to participate less in leisure and social activities throughout childhood.

The positive impact of physical exercise reducing ASD symptomology and the associated comorbidities has been sufficiently explored. Several systematic reviews and meta-analyses focused on synthesizing the effects of physical activity and sports participation in this population (Hume et al., 2021). Potential benefits noted in the literature include how physical activity can contribute to improvements in the social relations and communication skills of those with ASD (Bremer et al., 2016; Howells et al., 2019; Chan et al., 2021), how exercise can help reduce stereotyped behaviors (Ferreira et al., 2019), and the broad range of improvements in motor skills, social skills, and physical fitness resulting from these types of interventions (Sowa and Meulenbroek, 2012; Sam et al., 2015; Healy et al., 2018).

Participating in combat sports can help improve the physical and mental health of children with ASD, particularly when it comes to improving participants’ motor skills (Kim et al., 2016; Sarabzadeh et al., 2019), but there are also studies evaluating the benefits in social terms. For example, researchers have shown that karate training can significantly reduce stereotyped behaviors and improve social interaction (Bahrami et al., 2012; Movahedi et al., 2013). It is worth highlighting that martial arts and combat sports training requires moderate-to-vigorous physical activity and, simultaneously, the need for concentration and self-control (Garcia et al., 2019). These sports may also appeal to young people with ASD because of the repetitive nature of the tasks involved in the training (Bell et al., 2016).

Among martial arts, the specific characteristics of judo, with its alternating phases of vigorous physical activity and low-intensity exercises focused on mindfulness, may provide an ideal method to reduce the stress response (e.g., cortisol levels) of young people with ASD (Renziehausen et al., 2022). Indeed, a recent systematic review (Pečnikar Oblak et al., 2020) highlights the health and psychosocial benefits that people with mental disabilities can gain from participating in adapted judo programs. Short-term judo programs have reduced repetitive behaviors and improved social communication, interaction, and emotional responsiveness (Morales et al., 2021). At the same time, a study with an eight-week intervention (Rivera et al., 2020) found a decrease in aggressive behaviors among children with ASD who had taken part in an adapted judo program. Other researchers have demonstrated the viability and effectiveness of these programs, observing that the participants tend to embrace and enjoy adapted judo and express a desire to continue participating (Tomey, 2017). Adapted judo programs have registered good adherence rates leading to overall increases in moderate-to-vigorous physical activity (Garcia et al., 2019). However, the impact of participation in extended-duration judo training beyond 8 weeks in children with ASD has yet to be fully explored.

In light of this evidence of the effectiveness of adapted judo programs for people with ASD, the main objective of this study is to determine the effects of a long-term adapted judo program on the motor skills and psychosocial behaviors in children with ASD. The secondary objective is to observe the relationship between motor skills and the severity of psychosocial behavior symptoms of children with ASD. The hypothesis is that the participants in the adapted judo program will improve the motor skills and psychosocial behaviors assessed in the study.

## Materials and methods

### Participants

This study featured the participation of 40 children with a mean age of 11.07 (±1.73) years, a mean height of 145.9 (±15.81) cm and a mean weight of 47.71 (±16.71) kg. A sample description by group is presented in Table 1. The participants were recruited with the assistance of several different associations of families of people with ASD and schools for children with special needs. All the participants had been diagnosed with ASD according to the criteria of the Diagnostic and Statistical Manual of Mental Disorders – Fifth Edition (DSM-5). The psychological reports provided by the participants indicated that their intelligence quotients (IQ) ranged from 55 to 70 (mean value 61.4 ± 3.55). Exclusion criteria included individuals who had been advised not to participate in physical exercise, those who had previously taken judo lessons and those who were already participating in extracurricular athletic activities. Participation was voluntary, and the participants and their families were verbally and in writing informed of the program’s characteristics. The participant’s parents, legal guardians, and children signed an informed consent document explaining the program’s plan and objectives. All the protocols within this research, including the treatment of the participant’s personal information, followed the 1975 Declaration of Helsinki requirements and the subsequent revisions. This study received the approval of the Research Ethics Committee of Universitat Ramon Llull under file number CER URL_2019_2020_003, and the trial was registered on Clinicaltrials.gov (NCT04523805).

**Table 1:** Sample characteristics by group.

Variable	Experimental group (*n* = 21) Mean (standard deviation)	Control group (*n* = 19) Mean (standard deviation)
Age (years)	10.82 (±1.6)	11.35 (±1.9)
Height (cm)	143.91 (±13.01)	147.95 (±16.45)
Weight (kg)	45.96 (±17.09)	49.67 (±12.01)
IQ	60.8 (±3.05)	61.9 (±4.13)

### Procedure

The present study was prospective and employed a convenience sampling method. The sample was divided into two groups according to their willingness and commitment to participate in an adapted judo program over a school year. Thus, the sample consisted of an experimental group (*n* = 21) that took part in a 6-month adapted judo program and a control group (*n* = 19) that did not participate in extracurricular sports over this period. The experimental group participated exclusively in the adapted judo program without the possibility of regular participation in other sports activities, which could bias the results. Each participant was assessed twice, at the beginning and the end of the program, under stable conditions and in the same room where the judo sessions were held.

### Intervention

The experimental group took part in a 6-month adapted judo program. The judo sessions were performed in a large, well-ventilated, and safe space suitable for judo practice. The judo mat had a surface area of 120 m^2^ and was made of high-density foam designed to reduce the impact of falls, ensuring safe practice. Each participant was outfitted with a *judogi* (the judo suit consisting of a cotton jacket, trousers, and a belt).

Participants completed one session per week of 90 min of duration. Two judo teachers with 7th and 6th degree black belts, with academic backgrounds in pedagogy and sports sciences, respectively, led the sessions, and at least four volunteer judo instructors lent support. The sessions consisted of judo-specific tasks preceded by a warm-up and ending with a cool-down. As previously employed in other studies of our research group (Morales et al., 2021), the judo-specific content of the sessions included:

- Different types of general movements and falling techniques (from stepping in all directions to body repositioning and turning, movements from stable to unstable supports).
- Simplified judo-specific movements and games (building up body contact through games, primary focus on essential/simplified judo movements).
- Body control techniques on the ground and throws (progression of techniques from simplified to more complex movements).
- Repetitions of basic technical movements in different directions (pulling, pushing, holding, lifting).

The method of instruction utilized the principle of gradual progression, ensuring the consolidation of concepts learned in the initial lessons before moving on to more complex activities and material. Each participant progressed at his or her own pace.

The judo program was adapted by applying the principle: “normal where possible, adapted where necessary” (Morales et al., 2022). The main attributes of our adapted judo program were:

- The learning method chosen was imitation. The instructor presented techniques and then guided the practice.
- The employment of very marked routines based on brief and clear instructions in the form of the five “W” (who, when, what, where, why).
- Judo teachers spoke in a calm and firm voice. They gave objective instructions and refrained from using figurative language or irony.
- It can be deduced that sometimes it can be difficult for people with ASD to see the big picture because they perceive so many details.
- Instructors were trained to keep calm and not criticize the slowness of reproducing movements that sometimes characterize people with ASD.
- Spontaneous and unexpected behavior changes were monitored and redirected by judo teachers. They were aware that each participant required their own time.
- Instructions were given repetitively and employed a broad spectrum of senses, not only verbal cues. The isolated use of sensory instructions, one at a time, can support perception. For example, the instructor may physically demonstrate with verbal instructions and once without speaking.

### Assessment instruments

All participants completed two assessment sessions, once at the start of the intervention and again 6 months later, with the Gilliam Autism Rating Scale-Third Edition (GARS-3; Gilliam, 2014) and the Test of Gross Motor Development (TGMD-3; Ulrich, 2019).

The TGMD-3 is an instrument designed to assess the gross motor performance of children from 3 to 10 years of age. The assessment includes subscales measuring Locomotor Skills and Ball Skills, representing the fundamental motor skills most commonly taught in physical education classes worldwide. This study used a visual support protocol previously validated for children with ASD (Allen et al., 2017), whereby the instructors explained the test elements *via* a combination of illustrated cards, verbal instructions and physical demonstrations.

In addition to detecting delays and limitations in children’s gross motor skill development, the TGMD-3 can also be used as a research tool to explore gross motor skills, both among children with a typical development pattern and among those with atypical movement functioning (Ulrich, 2019). The Locomotor Skills subscale features six movements that require coordination and movements in various directions (Run, Gallop, Hop, Skip, Horizontal jump, Slide). The Ball Skills subscale assesses throwing, hitting and catching skills through seven tasks: Two-hand strike, One-hand strike, Dribble, Two-hand catch, Kick, Overhand throw, and Underhand throw. The test is video recorded and later analyzed by trained independent (blinded) raters using a screening tool and the criteria set out in the TGMD-3 for each task. Participants are assigned a score of “1” for each task they successfully completed and a score of “0” when they do not meet the criteria. After this analysis, the locomotor and ball skills subscale scores are summed, yielding a raw total TGMD-3 score.

The GARS-3 is an instrument used to assess changes in the severity of ASD behaviors. As described in a previous work of our research group (Morales et al., 2021), the GARS-3 includes 56 items describing characteristic behaviors of individuals with ASD. The items are grouped into six subscales: repetitive behaviors (RB), social interaction (SI), social communication (SC), emotional responses (ER), cognitive style (CS), and maladaptive speech (MS). Parents and caregivers had to score each item on a four-point Likert-type scale (0 = never observed; to 3 = frequently observed) spending approximately from 5 to 10 min, and based on the frequency of occurrence of each item under ordinary circumstances in a 6-h period. The raw scores for each subscale were summed, yielding a total GARS-3 score. Both the pre-and post-tests were carried out using pen and paper. Parents and caregivers had the opportunity to ask questions about item interpretation.

### Statistical analysis

All descriptive data from the dependent variables are presented with the mean ± standard deviation (SD). The normality of the distribution of each variable was checked with a Shapiro-Wilks test. A one-way repeated measures MANOVA evaluated the effects of an intra-subject factor (TIME: pre-post) and an inter-subject factor (GROUP: control-experimental) of each dependent variable.

Multivariate contrast monitoring was performed using univariate contrast to determine any significant differences in dependent variables between conditions. The effect size of the multivariate and univariate contrasts was calculated using the partial eta squared (*η^2^p*) and interpreted as a small, medium, or large effect when the values of *η^2^p* reached 0.0099, 0.0588, and 0.1379, respectively (Cohen, 1988). When univariate contrasts showed statistically significant interaction effects, pairwise comparisons with Bonferroni correction were applied.

**Figure 1:**
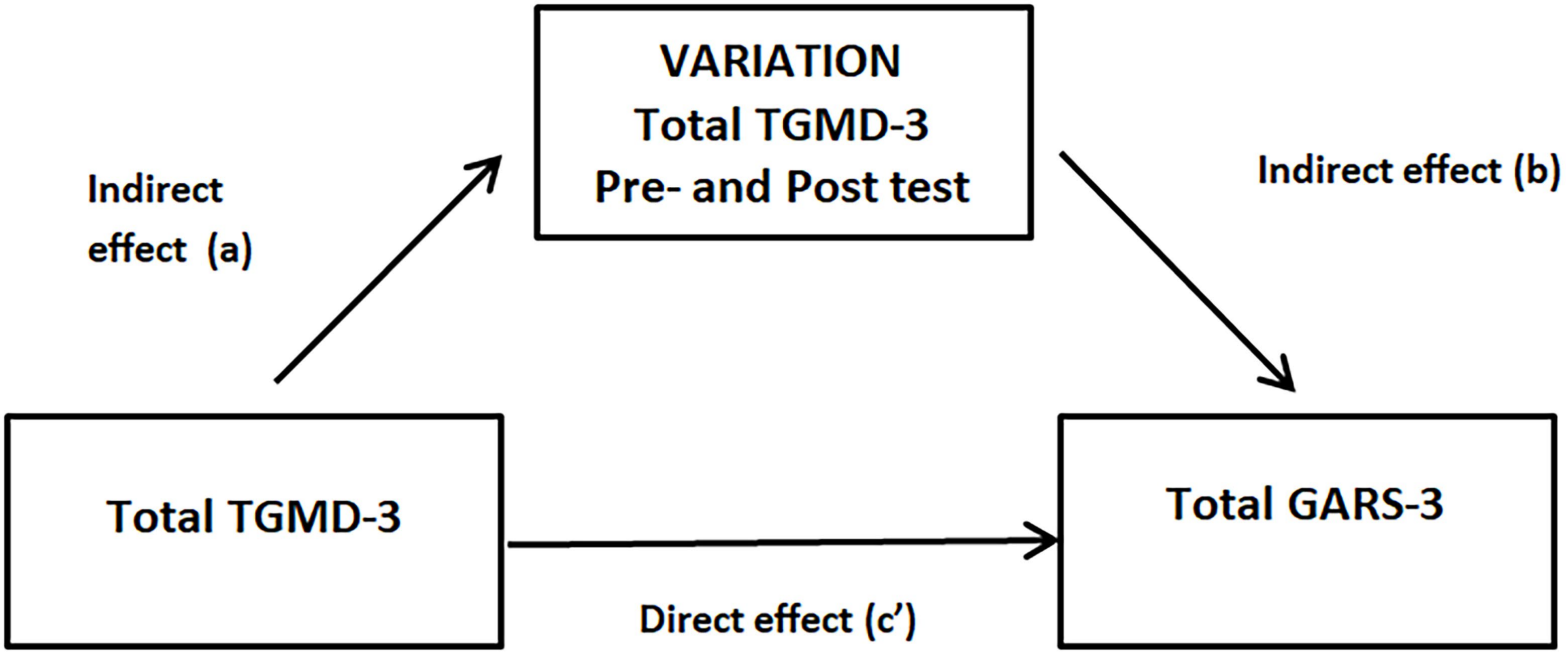
Mediation analysis. Contribution of total TGMD-3 post-test scores on total GARS-3 post-test scores through the variation in the total GARS-3 score (calculated as the difference between the pre-and post-test scores). TGMD-3 = Test of Gross Motor Development-Third Edition; GARS-3 = Gilliam Autism Rating Scale-Third Edition.

A simple mediation analysis model measured the relationship between motor skills and the severity of psychosocial behaviors among children with ASD. This simple mediation analysis examined motor skills’ direct and indirect effects using the total TGMD-3 post-test scores as a predictive variable (PV) of the total GARS-3 post-test scores, representing the dependent variable (DV) of psychosocial behaviors. This analysis used the variation in the total GARS-3 score (calculated as the difference between the pre-and post-test scores) as a mediating variable (MV). The associations explored in this model are represented in Figure 1.

A simple mediation analysis indicates a direct effect (c) when the PV exerts an influence on the DV without taking into account the analysis of the participation of the MV. Meanwhile, the model indicates an indirect effect (a and b) when the PV exerts its influence on the DV through the MV. Finally, a total effect is registered when the PV influences the DV in the presence of the MV but not *via* this mediating variable.

All the statistical analyses were calculated using the Statistical Package for Social Science version 24.0 software (SPSS, Inc., Chicago, IL, United States). A significance level of *p* < 0.05 was used for all tests. (Data available at https://doi.org/10.6084/m9.figshare.20465337).

## Results

All the dependent variables did not show significant differences at the pre-test between the control and experimental groups, indicating that they were homogeneous groups at the beginning of the intervention.

**Figure 2:**
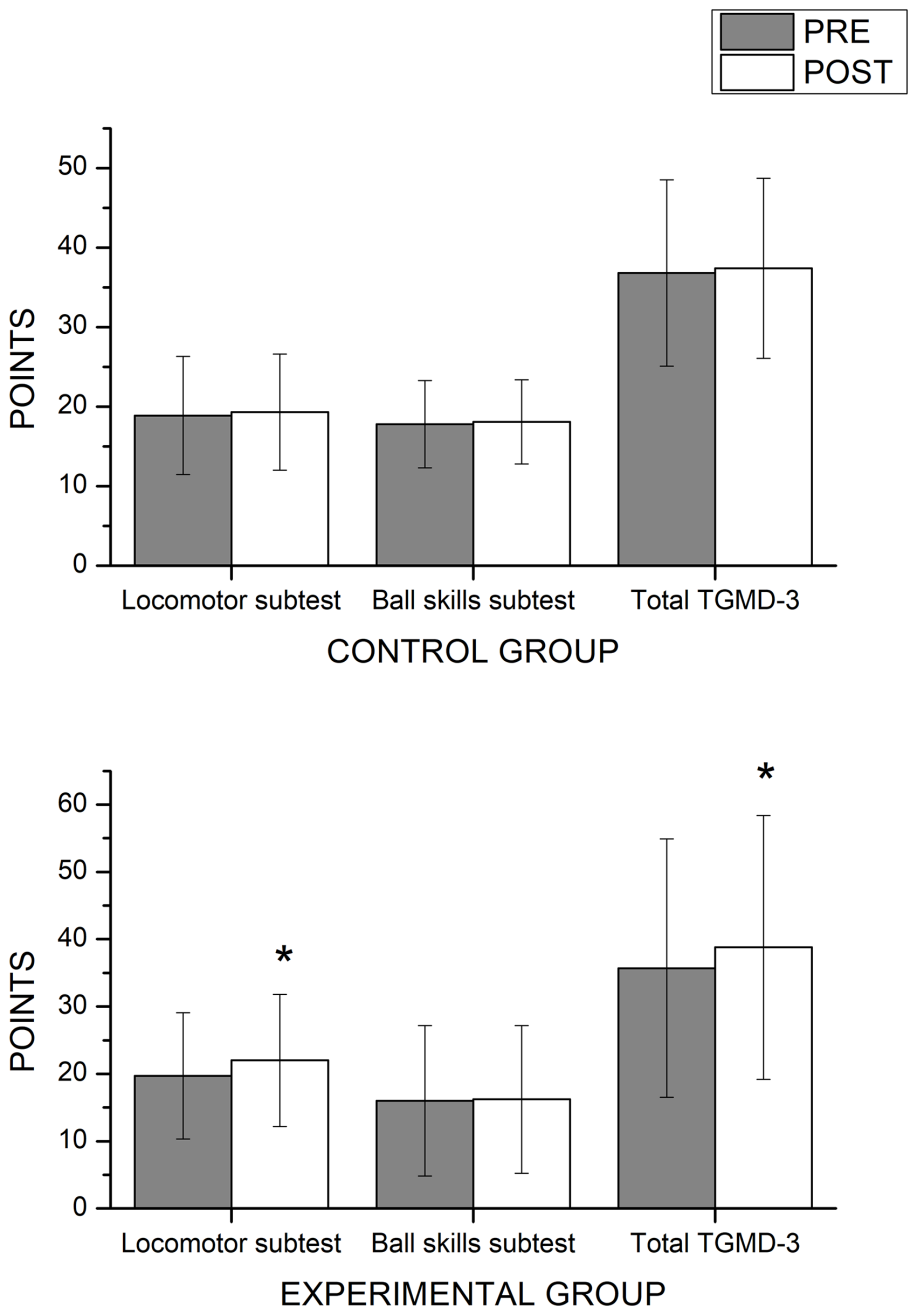
Pre- and post-scores in Total TGMD-3 and its Locomotor and Ball Skills subscales after 6 months adapted judo program by group. *Significant differences (*p* < 0.05).

The multivariate analysis applied to the outputs of the TMGD-3 test showed a significant time × group interaction (*F*_3,43_ = 10.18, *p* < 0.001, *η^2^p* = 0.41) and a significant effect of time (*F*_3,43_ = 23.08, *p* < 0.001, *η^2^p* = 0.61). Follow-up of the univariate analysis in this test showed an interaction effect (time × group) in the Locomotor Skills subscale score (*F*_1,42_ = 27.87, *p* < 0.001, *η^2^p* = 0.38) and the total TGMD-3 score (*F*_1,42_ = 20.06, *p* < 0.001, *η^2^p* = 0.31). The Ball Skills subscale did not show interaction effect. Finally, we observed significant improvements in the experimental group between the pre-and post-test in the Locomotor Skills subscale (*p* < 0.001) and Total TGMD-3 (*p* < 0.001) (Figure 2).

**Figure 3:**
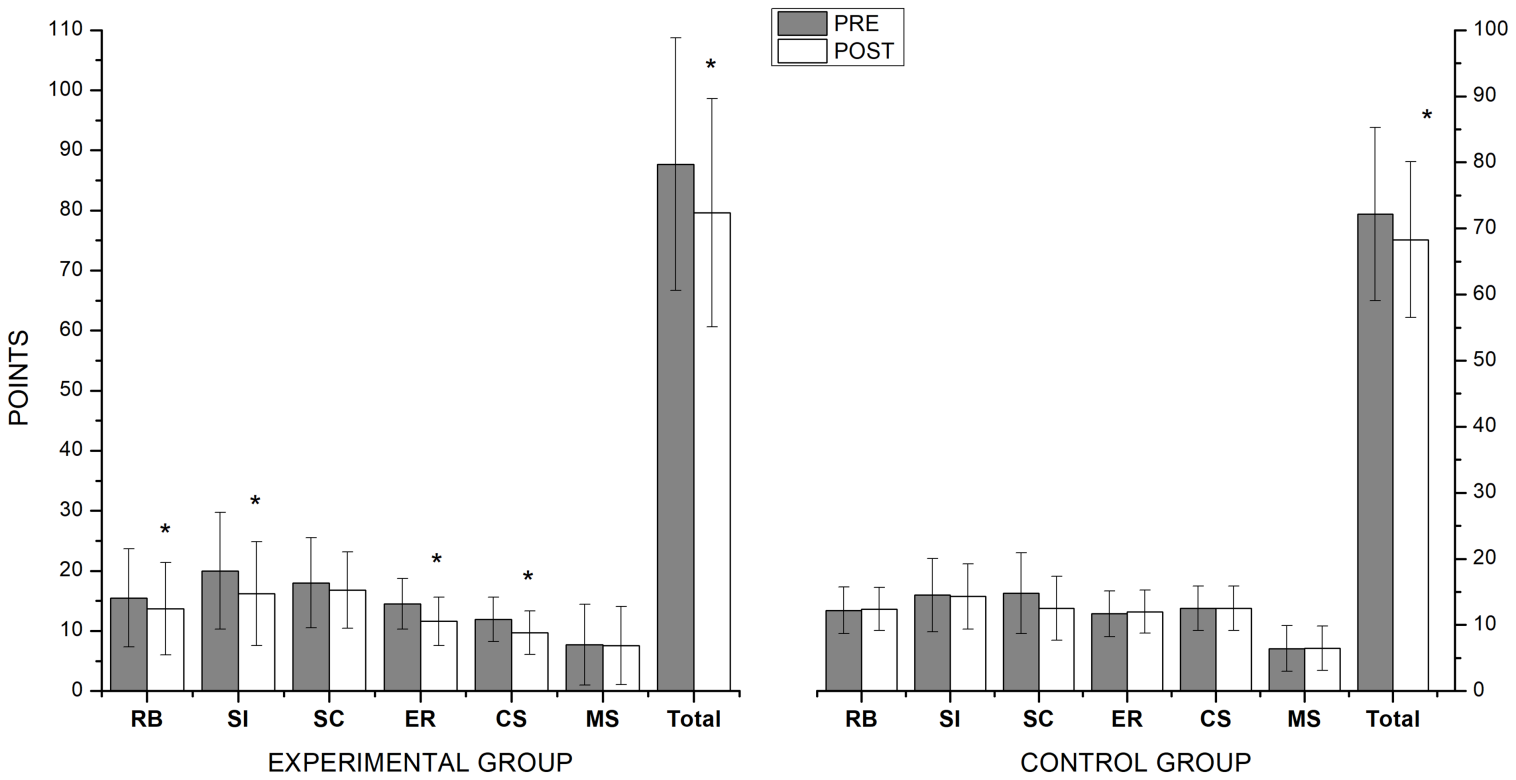
Pre- and post-scores on the Gilliam Autism Rating Scale-Third Edition (GARS-3) subscales forrepetitive behaviors (RB), social interaction (SI), social communication (SC), emotional responses (ER), cognitive style (CS), and maladaptive speech (MS) after 6 months of adapted judo program intervention. *Significant differences (*p* < 0.05).

The multivariate analysis applied to the outputs of the GARS-3 test showed a significant time × group interaction between (*F*_7,32_ = 31.25, *p* < 0.001, *η^2^p* = 0.87) and a significant effect of time (*F*_7,32_ = 12.06, *p* < 0.001, *η^2^p* = 0.72). Follow-up of the univariate analysis in this test showed an interaction effect (time × group) in the RB subscale score (*F*_1,38_ = 6.29, *p* < 0.016, *η^2^p* = 0.14), SI subscale score (*F*_1,38_ = 60.39, *p* < 0.001, *η^2^p* = 0.61), ER subscale score (*F*_1,38_ = 8.40, *p* < 0.006, *η^2^p* = 0.18), CS subscale score (*F*_1,38_ = 4.20, *p* < 0.046, *η^2^p* = 0.10) and the total GARS-3 score (*F*_1,38_ = 17.39, *p* < 0.001, *η^2^p* = 0.31). The SC and MS subscales did not show interaction effect. Finally, the pairwise comparison showed significant improvements in the experimental group between the pre and post-test in the RB (*p* = 0.01), SI (*p* < 0,001), ER (*p* = 0.002) and CS (*p* = 0.005) subscales, and Total GARS-3 (*p* < 0.001). (Figure 3).

The results of the simple mediation analysis (Table 2) showed a significant total effect when the PV (total TMGD-3 score) influences the DV (total GARS-3 score) in the presence of the MV (GARS-3 variation), but not through the latter variable. This result demonstrates that motor skills performance is closely linked to the severity of ASD in terms of psychosocial behaviors and that the variation in motor skills recorded during the intervention influenced this relationship, albeit a small one, because the values for the variation include the control group.

**Table 2:** The approximate mediating role of the variation of motor skills in the relationship between psychosocial behaviors and motor skills.

Effect	Coefficient	95% Confidence interval	*Z*	*p*-value	% mediation
Indirect (a × b)	0.009	−0.01; 0.02	1.00	0.316	3.76
Direct (c)	0.243	−0.34; −0.14	4.64	< 0.001*	96.24
Total (c + a × b)	0.234	−0.33; −0.13	4.32	< 0.001*	100.0

*p < 0.05 indicates statistically significant differences.

## Discussion

This study offers a complete description of the effects of a long-term adapted judo program on children with ASD and illustrates the psychosocial behavioral changes that occurred in the sample during the intervention.

The study’s results confirm the initial hypothesis, as most indicators showed improvements in the experimental group’s scores after participating in the adapted judo program. After the intervention, the children in the experimental group recorded improvements in motor skills, as measured by the total score on the TGMD-3 and the Locomotor Skills subscale. Meanwhile, the children in the control group did not display any significant changes. The experimental group also showed significant improvements in the total GARS-3 score and on the subscales measuring RB, SI, ER, and CS.

It has been established that children with ASD tend to engage in sedentary activities and generally are less physically active than their neurotypical peers during their free time (Zhao and Chen, 2018). As such, guided activities like the judo sessions described within this study might be needed to encourage the participation of children with ASD in physical activity. In fact, some prior studies of short-term adapted judo programs for children with this disorder have achieved high adherence among participants, increased moderate-to-vigorous physical exercise (Garcia et al., 2019), and improved satisfaction and self-confidence (Tomey, 2017).

We have been unable to find other studies that have examined the improvement of the motor skills of children with ASD through judo. There are, however, some examples of research using other martial arts disciplines or other combat sports, such as a case study observing the balance and postural control benefits of an intervention program based on aikido (Polak et al., 2019) and another eight-week intervention using taekwondo (Kim et al., 2016). Systematic participation in judo requires balance and coordination tasks as well as the development of grip strength (Demiral, 2011). In addition to the usual elements of judo, the adapted program that was applied in this study included a large number of judo-specific movement exercises that coincided with the tasks assessed on the Locomotor Skills subscale of the TGMD-3, meaning that it was reasonable to expect the improvements that were achieved in this regard. Meanwhile, there is evidence that participation in judo can also help improve motor skills such as agility, coordination and balance among neurotypical children (Protic-Gava et al., 2019; Purnamasari et al., 2021). Another study featuring a year-long judo program in children found improvements in various domains of motor skills and physical fitness, such as hand strength, flexibility and overall coordination (Toskić et al., 2014), while judo training has been shown to improve body posture, balance and lower limb muscle strength (Walaszek et al., 2017).

Meanwhile, Lo et al. (2019) have shown that using judo in school can improve spatial orientation skills. These researchers recommended using judo with people with ASD in order to improve their executive functioning. Our results echo these findings, as the participants showed improvements in postural control and spatio-temporal orientation (Run, Gallop, Hop, Skip, Horizontal jump, Slide) following the intervention. Additionally, a high degree of correlation was found between motor skills and executive functioning, suggesting a relationship between these two constructs and those interventions aimed at one of these two areas might also impact the other (Hilton et al., 2014). In light of this relationship between motor skills and executive functioning, improvements in the former might help children with ASD in their everyday activities and, in broader terms, contribute to increasing their quality of life.

After participating in the long-term adapted judo program, the children with ASD significantly reduced psychosocial behavioral ratings. The results are similar to those found in our previous work following an eight-week intervention (Morales et al., 2021). In both cases, significant improvements were recorded on six of the subscales of the GARS-3, although the list of scales with significant differences was not the same in the two studies. There were no significant differences in the subscale measuring maladaptive speech in either of the studies, which might indicate that participating in sports does not tend to improve the language skills of people with ASD, as previously reported following an intervention involving horseback riding (Gabriels et al., 2015). Compared to the earlier short-term judo intervention, one unique finding of the current study is that the Communication subscale did not display significant improvements. This result stands in contrast with the general trend reported in systematic reviews and meta-analyses of physical activity and sports participation interventions (Bremer et al., 2016; Healy et al., 2018; Howells et al., 2019), which have observed improvements in communication skills presumably due to the experiences of teamwork and social interaction.

The overall results regarding psychosocial behaviors coincide with previous investigations following short-term adapted judo programs, which have been found to decrease stress, increase satisfaction with the activity, and improvements in social relations (Rivera et al., 2020; Renziehausen et al., 2022). These findings are further confirmed by other experiences with neurotypical people that have found that participating in judo increases empathy, reduces aggressive behavior (Ennigkeit and Beek, 2019) and contributes to the development of self-discipline, serenity, efficient problem solving and socio-moral sensitivity (Sterkowicz-Przybycień et al., 2014). It is also worth highlighting the successful record of adaptations of other martial arts in reducing stereotyped behaviors and improving social interactions within this population (Bahrami et al., 2012; Movahedi et al., 2013; Phung and Goldberg, 2019). Combat sports and martial arts like judo have clear structures and other elements that make them very suitable for people with ASD and are likely to contribute to improvements in various aspects of their everyday lives. These kinds of sports can be adapted to the individual characteristics of each participant and can be applied in different contexts, such as various exercise intensities that might cater to children with ASD. Additionally, these sports are highly structured and involve repetitive movements that are easily mimicked and mental imagery with opponents (Bahrami et al., 2012). One possible reason for the effectiveness of the adapted judo program was that it was designed according to some of the traditional tenets of martial arts training. In other words, it extended beyond physical activity, with additional emphases on self-discipline and behavioral, emotional and cognitive control, as previously recommended for combat sports and martial arts interventions for children with ASD (Morales et al., 2021).

Concerning the secondary objective of this study, the results showed a significant relationship between the variables measuring motor skills and those measuring psychosocial behaviors. The participants’ total TGMD-3 scores directly affected the total GARS-3 score, with the mediation calculated at 96.24%. The same mediation analysis also showed a significant total effect, as determined by the mediation effect calculated by adding the direct effect and the indirect effect represented by the variation in scores on the TGMD-3 during the intervention period. These results suggest that motor skills and psychosocial behaviors are closely linked and that interventions addressing motor skills may influence this relationship to an extent.

There is considerable evidence showing that children with ASD experience greater difficulties with motor development than their peers (Miyahara, 2013). MacDonald et al. (2014) found a correlation between gross and fine motor skills and the severity of ASD. Specifically, they observed that more severe cases of ASD were associated with poorer fine and gross motor skills. Beyond delays in motor development among children with ASD, researchers have found links between these motor problems and the development of language and cognitive abilities (Bedford et al., 2016) and adaptive conduct (MacDonald et al., 2013), as well as between motor development and social skills (MacDonald et al., 2013).

One question that regularly emerges within the scientific literature on this topic is whether motor difficulties should be viewed as a central characteristic of ASD and whether this disorder should be co-diagnosed along with Developmental Coordination Disorder (DCD; Camden et al., 2022). Sumner et al. (2016) examined the relationship between motor skills and social abilities in a group of people with ASD, another with DCD and a control group in children, finding a good deal of overlap between the assessments of the motor and social skills of the ASD and DCD groups, both of which recorded lower scores than the control. Additionally, motor skills were found to predict social functioning in both of these groups.

Fundamental motor skills are the building blocks in developing more complex gross motor movements. If the intervention described here successfully improved motor skills, then the participants should be inspired to take a more positive view of their own motor abilities. This, in turn, might encourage them to continue participating in judo, as has been the case with other judo programs for people with ASD (Tomey, 2017; Garcia et al., 2019). Judo programs could also positively affect their social functioning and physical health. Importantly, it could also help them develop the motor skills needed to participate in other sports and physical activity programs with their neurotypical peers (Colombo-Dougovito and Block, 2019).

In childhood, free play is an opportunity to develop a range of motor skills. There does not seem to be any reason why children with ASD should not participate in play as neurotypical children do, but starting at the age of two the former tend to reduce their free play interactions (Serrada-Tejeda et al., 2021). However, they continue to display interest in directed play. In addition to offering directed activities, it is important to strive to adopt an inclusive approach to increase their effectiveness in physical activity interventions (Sansi et al., 2021). This could help address the difficulties that people with ASD often have in finding activities that meet their needs. Some of the motor development problems experienced by children with ASD might be due to this lack of opportunity. Adapted sports programs like the one described here are useful because they are designed specifically for the enjoyment of children with ASD and offer them the chance to contact other children. The characteristics of judo training mean that there is a combination of peer learning, which helps improve the relationships among the students with ASD, and guided activities where they learn the technique of the sport by imitating their instructors. Finally, the structure of judo sessions and the routines associated with the sport, such as formal procedures, including bowing, might help the participants overcome some of the difficulties they face in their everyday lives and at school.

This study may have some limitations because the data was collected from non-blinded caregivers, which was the case for the GARS-3. This can be a source of confusion in this type of intervention-based research, as it can be unclear whether the knowledge of the intervention by the caregivers could influence their answers or whether the results could be affected by subjective factors such as the expectations involved with the adapted judo program. In any case, the GARS-3 protocol recommends that the people most familiar with the participants’ behavior complete the questionnaire. Furthermore, the sampling distribution within groups was not randomized. As stated in the methods section, they were distributed into two groups according to their willingness and commitment to participate in an adapted judo program over a school year. The lack of randomization could have biased the results; nevertheless, experimental and control groups presented similar values in all the dependent variables at the pre-test, indicating that they were homogeneous groups at the beginning of the intervention.

## Conclusion

The most important conclusions of this study are that participating in a 6-months adapted judo program improved the motor skills and psychosocial behaviors of children with ASD. We can also conclude that there is a close relationship between motor skills and psychosocial behaviors, as the children with greater severity of autism-related behaviors were likely to display poorer motor skills. Meanwhile, the adaptation of the judo program increases its efficiency and could help participants adapt better to their everyday lives and improve their quality of life. In the present work, we have presented judo-specific content and instructional cues that could help apply the judo program to young people with ASD. Future research is warranted to study the effects of adapted judo programs in adults with ASD or other intellectual disabilities.

## Data availability statement

The original contributions presented in the study are publicly available. This data can be found at: https://doi.org/10.6084/m9.figshare.20465337.

## Ethics statement

The studies involving human participants were reviewed and approved by the Research Ethics Committee of Universitat Ramon Llull under file number CER URL_2019_2020_003, and the trial was registered on Clinicaltrials.gov (NCT04523805). Written informed consent to participate in this study was provided by the participants’ legal guardian/next of kin.

## Author contributions

JM and EC conceptualized the study, analyzed the data, and wrote the manuscript. EP assisted with conceptualizing the study, writing, and editing the manuscript. DHF assisted with data collection and editing the manuscript. VG assisted with data collection, writing, and editing the manuscript. MG-B assisted with data collection, interpretation of the results, writing, and editing the manuscript. MS-S assisted with interpretation of the results, writing, and editing the manuscript. All authors contributed to manuscript revision, read, and approved the submitted version.

## Funding

This study has been partially funded by a Ramon Llull University grant (ref. CER-URL-2019) and Erasmus+ Sport Programme (Project Identifier: 612954-EPP-1-2019-1-ES-SPO-SCP).

## Conflict of interest

The authors declare that the research was conducted in the absence of any commercial or financial relationships that could be construed as a potential conflict of interest.

## Publisher’s note

All claims expressed in this article are solely those of the authors and do not necessarily represent those of their affiliated organizations, or those of the publisher, the editors and the reviewers. Any product that may be evaluated in this article, or claim that may be made by its manufacturer, is not guaranteed or endorsed by the publisher.
